# Cerebrospinal Fluid Markers of Synaptic Injury and Functional Connectivity in Alzheimer Disease: Protocol for a Cross-Sectional Study

**DOI:** 10.2196/14302

**Published:** 2019-07-17

**Authors:** Rawan Tarawneh

**Affiliations:** 1 Department of Neurology College of Medicine The Ohio State University Columbus, OH United States

**Keywords:** Alzheimer disease, aging, functional imaging, synaptic injury, cerebrospinal fluid

## Abstract

**Background:**

Synaptic loss is the best surrogate for cognitive decline in Alzheimer disease (AD) and is more closely associated with cognitive function than amyloid or tau pathologies. Neurogranin (Ng) and synaptosome–associated protein-25 (SNAP-25) have demonstrated utility as cerebrospinal fluid (CSF) markers of synaptic injury in presymptomatic and symptomatic AD. While these synaptic markers have been shown to correlate with cognitive impairment and whole brain or regional atrophy in previous studies of AD, to our knowledge, the relationship between fluid markers of synaptic injury and functional brain imaging has not been previously investigated.

**Objective:**

The main objective of this study is to examine the relationship between CSF markers of synaptic injury (Ng and SNAP-25) and functional connectivity (FC) in the default mode and semantic memory networks in individuals with mild cognitive impairment (MCI) and mild dementia due to AD (Clinical Dementia Rating [CDR] 0.5-1) and cognitively normal controls (CDR 0), adjusting for age, gender, and the apolipoprotein E4 (APOE4) genotype. Secondary objectives include investigating the associations between CSF markers of amyloid and tau pathology (CSF tau, p-tau181, and Aβ42) and FC in the default mode and semantic memory networks in AD (CDR 0.5-1) and controls (CDR 0), adjusting for age, gender, and the APOE4 genotype.

**Methods:**

This is a cross-sectional study of individuals with MCI or mild dementia due to AD (CDR 0.5-1; n=20), and cognitively normal controls (CDR 0; n=20). Participants will undergo detailed clinical and neuropsychological assessments, CSF biomarker assessments (CSF Ng, SNAP-25, tau, p-tau181, and Aβ42 levels) and functional magnetic resonance imaging assessments, using a Siemens 3.0 Tesla Prisma scanner, during resting state and during the performance of a semantic memory task. All study procedures will be completed within 4 months of enrollment. Partial correlation analyses will examine associations of CSF biomarker measures with FC in the default mode and semantic memory networks in AD and controls.

**Results:**

This study was funded by the Chronic Brain Injury Discovery Themes of the Ohio State University College of Medicine. Study enrollment began in April 2018. Study procedures and data analysis are currently underway. Results are expected by December 2019.

**Conclusions:**

Findings from this study will further support the utility of CSF Ng and SNAP-25 as markers of synaptic injury by examining their associations with functional alterations in cortical networks affected by early AD pathology.

**International Registered Report Identifier (IRRID):**

DERR1-10.2196/14302

## Introduction

### Background

Amyloid plaques and neurofibrillary tangles are the two main pathological hallmarks of Alzheimer disease (AD) [1]. While amyloid and tau deposition begins a decade or more prior to the first signs of memory loss, it is only after significant neuronal and synaptic loss has occurred in vulnerable brain regions that the first signs of cognitive impairment appear [2]. Pathological studies of AD and proposed models of disease progression suggest that synaptic loss is the best surrogate for cognitive decline in AD [3,4] as it appears to be more closely associated with cognitive outcomes than the degree of amyloid plaques, neurofibrillary tangles, or gliosis in AD brains [5].

Neurogranin (Ng) and synaptosome–associated protein-25 (SNAP-25) have recently been identified as potential cerebrospinal fluid (CSF) biomarkers of synaptic injury in AD [6,7]. Ng and SNAP-25 are synaptic proteins which are abundantly and preferentially expressed in the presynaptic (SNAP-25) or postsynaptic (Ng) membranes and are widely distributed in the human brain [8,9]. Ng is a neuron-specific [8] calmodulin-binding postsynaptic protein [10] which is abundantly expressed in neuronal dendritic spines. Several studies have implicated Ng in activity-dependent synaptic plasticity, memory, and learning [10-14]. Ng enhances synaptic function [8] and facilitates long-term potentiation by regulating the availability of calmodulin at synaptic sites [15-17]. SNAP-25 is a widely distributed presynaptic protein which is involved in docking and fusion of synaptic vesicles, a process essential for exocytosis [18]. SNAP-25 has also been implicated in axonal outgrowth and neurite elongation [19].

Studies by our group and others have shown that CSF Ng [7,20] and SNAP-25 [9,21] levels are elevated in AD compared to controls. Elevated CSF levels of synaptic proteins in AD likely reflect the release of abundant synaptic constituents into the extracellular space in the setting of neurodegeneration [7]. We have previously shown that CSF Ng levels strongly correlate with CSF levels of tau and tau phosphorylated at threonine 181 (p-tau181), whole brain and regional atrophy, and rates of cognitive decline in a large, well-characterized cohort of cognitively normal individuals and individuals with presymptomatic and early symptomatic AD. These individuals were enrolled in studies of aging and dementia at the Knight Washington University Alzheimer’s Disease Research Center and followed longitudinally for 2-3 years [7]. Furthermore, we have shown that CSF Ng levels correlate with cortical amyloid deposition in presymptomatic AD [7]. Importantly, in the previous cohort, CSF Ng offered a predictive value for future cognitive impairment in cognitively normal individuals over a 2-3 year follow-up period that was comparable to other biomarkers of AD pathology (CSF tau, p-tau181, and Aβ42). CSF Ng also complemented the collective ability of these markers to predict AD pathology in cognitively normal elderly individuals (ie, presymptomatic AD). Data from our group [22] and others [9,21], suggest that CSF SNAP-25 offers value as a diagnostic and predictive marker in early AD, and correlates with other CSF biomarkers of AD pathology. Together, these findings support the value of CSF Ng and SNAP-25 as CSF surrogates of synaptic injury in AD.

Functional magnetic resonance imaging (fMRI) studies have identified networks of cortical regions which demonstrate highly synchronized activity during the resting state or during the performance of specific cognitive tasks [23,24]. The default mode network (DMN), which includes the posterior cingulate, precuneus, medial temporal, medial prefrontal, and inferior parietal regions, is active during rest and shows reduced activity during cognitive tasks [25,26]. Reduced functional connectivity (FC) within the DMN has been shown in early AD, including mild cognitive impairment (MCI) due to AD [24,27,28].

Semantic memory refers to the recall of general facts and knowledge that are not contextually specific (eg, making a categorical or attributional judgment to a presented item) [29]. The neural correlates of semantic memory include a left lateralized network of cortical regions, including the posterior cingulate, precuneus, parahippocampal gyrus, posterior inferior parietal, middle temporal, fusiform, dorsomedial prefrontal, ventromedial prefrontal, and inferior frontal cortices [30,31]. Previous studies have shown the utility of the Famous Name Discrimination Task (FNDT) in evaluating FC within the semantic memory network in older adults, including those with early AD [32].

To our knowledge, no studies have investigated the utility of CSF Ng or SNAP-25 as surrogates of synaptic injury in functional imaging studies of healthy aging and AD. In this study, we propose to investigate associations between CSF Ng or SNAP-25 levels and FC measures in the default mode and semantic memory networks in cognitively normal older adults and those with early symptomatic AD, including MCI (Clinical Dementia Rating [CDR] 0.5) and mild dementia (CDR 1) due to AD. Findings from this study will support the utility of CSF Ng and SNAP-25 as fluid surrogates of synaptic injury in AD by evaluating their associations with functional imaging as an in vivo marker of synaptic integrity. CSF biomarkers that reflect functional alterations in neural networks targeted by early AD pathology (ie, default mode and semantic memory networks) will offer valuable tools to monitor disease progression and response to disease modifying therapies in clinical trials of AD therapeutics, independently of changes to amyloid or tau pathology, and will supplement information provided by cognitive and imaging outcome measures.

### Study Objectives

#### Primary Objectives

The first primary objective of the study is to investigate correlations between CSF biomarkers of synaptic injury (Ng and SNAP-25) and FC in the DMN using resting state fMRI (adjusting for age, gender, and the apolipoprotein E4 [APOE4] genotype) in AD (CDR 0.5-1) and controls (CDR 0). The second primary objective is to examine correlations between CSF biomarkers of synaptic injury and FC in the semantic memory network on task activated fMRI during the performance of the FNDT (adjusting for age, gender, and the APOE4 genotype) in AD and controls.

#### Secondary Objectives

This study has three secondary objectives. The first is to investigate potential correlations between established AD biomarkers (CSF tau, p-tau181, and Aβ42) and FC in the DMN using resting state fMRI (adjusting for age, gender, and the APOE4 genotype) in AD and controls. The second is to examine the possible correlations between established AD biomarkers and FC in the semantic memory network on task activated fMRI during the performance of the FNDT (adjusting for age, gender, and the APOE4 genotype) in AD and controls. Lastly, the third objective is to compare correlations of novel (CSF Ng and SNAP-25) and established AD biomarkers (either individually or in different combinations) with FC in the default mode and semantic memory networks in AD and controls.

## Methods

### Overview

This will be a cross-sectional study of individuals with MCI due to AD or mild AD dementia (CDR 0.5-1; n=20), and cognitively normal controls (CDR 0; n=20). All participants will undergo a detailed clinical and neuropsychological assessment during the first visit, one lumbar puncture (LP) during the second visit, and one structural and functional MRI assessment during the third visit (Table 1). Functional MRI data will be acquired during resting state and performance of a semantic memory task (ie, FNDT). Cognitive, CSF, and MRI assessments will be completed within 4 months of enrollment. Resting state and task activated fMRI scans will be conducted in the same setting to minimize effects of environmental factors on fMRI parameters.

### Participants

Participants will be recruited from the community and the Cognitive Neurology Clinic of the Ohio State University Wexner Medical Center. This study will include n=20 cognitively normal individuals (CDR 0), and n=20 individuals with a clinical diagnosis of single-domain or multi-domain amnestic MCI due to AD (CDR 0.5) or mild AD dementia (CDR 1). As some participants who enroll in the study may later elect to withdraw their participation (ie, drop out) or may be lost to follow-up, we anticipate the need to enroll a total of 50 participants (CDR 0, n=25; and CDR 0.5-1, n=25) to maintain adequate statistical power for the study.

#### Inclusion Criteria

Participants included in the study will be: 1) 60 years of age or older, with a clinical diagnosis of amnestic MCI due to AD or mild AD dementia, or with normal cognition (See Criteria for Diagnostic Classification); 2) will have no significant medical or surgical comorbidities; 3) will have no contraindications to LP or MRI (see Multimedia Appendix 1); and 4) will have adequate visual and auditory acuity for testing. In addition, participants must have a responsible study partner who either lives with them or is in regular contact with them for at least 10 hours per week.

#### Exclusion Criteria

Criteria which exclude participants from the study include: 1) MCI due to AD or mild AD dementia being treated with cholinesterase-inhibitors (CHEI) or memantine within 3 months of study enrollment, or with a dosage of these medications that has been adjusted in the 3 months prior to enrollment; 2) any past history of ischemic or hemorrhagic strokes; 3) traumatic brain injury (including concussions); 4) imaging evidence of significant cerebrovascular disease or structural brain lesions (eg, tumor, demyelinating disorders, or infection); 5) an active mood or psychiatric disorder; 6) active daily alcohol use; (7) past or current history of alcohol abuse or dependence; or 8) active, daily or frequent (≥2 times/week) use of benzodiazepines, barbiturates, anticholinergics, antihistamines, sedatives, sleep aids, or antiepileptic medications in the 3 months prior to study enrollment.

**Table 1:** Study overview and procedures.

Study visit	Evaluations and procedures to be completed	Estimated visit duration
1	A detailed clinical history, including a detailed review of the history of present illness, past medical, surgical, social, and family history, medications, and allergies from study participants and their study partners A detailed physical and neurological exam A detailed neuropsychological assessment which includes evaluation of verbal and nonverbal memory, language, attention, processing speed, executive and visuospatial functions in addition to behavioral and functional assessments Blood sample collection for APOE4^a^ genotyping and screening coagulation parameters A detailed review of eligibility criteria for the LP^b^ and MRI^c^ assessments (see Multimedia Appendix 1)	3-4 hours
2	Lumbar puncture (collection of 20-25 ml of cerebrospinal fluid)	60-90 minutes
3	MRI during resting state and performance of a semantic memory task (ie, Famous Name Discrimination Task)	90 minutes

^a^APOE4: apolipoprotein E.

^b^LP: lumbar puncture.

^c^MRI: magnetic resonance imaging.

Eligible participants who have been on stable doses of CHEI and/or memantine for ≥3 months at the time of enrollment, and who meet the other eligibility criteria for the study, will be included. Eligible study participants will be instructed to avoid the use of alcohol, benzodiazepines, over-the-counter sleep aids, antihistamines, and anticholinergic medications for at least 2 weeks prior to the time of their enrollment and for the whole duration of the study.

Written informed consent will be obtained from all participants or their legally authorized representatives when appropriate. Additionally, written informed assent will be obtained from all participants with mild AD dementia (CDR 1).

### Criteria for Diagnostic Classification

The clinical diagnosis of amnestic MCI due to AD will be made according to standard clinical criteria as described by the National Institute on Aging and Alzheimer’s Association (NIA-AA) Working Group [33]. Clinical diagnoses will be supported by CSF biomarker data for tau, p-tau181, and Aβ42 (ie, the CSF biomarker phenotype of AD including elevated CSF tau or p-tau181, and low CSF Aβ42 levels) at the time of data analysis. This includes evaluation for other systemic or neurological disorders which could significantly contribute to cognitive impairment, and inclusion of results from ancillary structural imaging (brain computed tomography [CT] or structural MRI), neuropsychological testing, and ^18^F-fludeoxy-glucose positron emission tomography (FDG-PET) imaging (when available) into the diagnostic scheme [33].

The diagnosis of amnestic MCI will be based on impairment in episodic memory with or without impairment in other cognitive domains (ie, multi-domain and single-domain amnestic MCI, respectively), which is 1-1.5 SDs of age-, gender-, and education-matched norms, and is not associated with significant functional decline [33]. In most centers, a diagnosis of amnestic MCI due to AD is equivalent to a CDR of 0.5.

The clinical diagnosis of dementia due to AD will be made according to standard clinical criteria as described by the NIA-AA Working Group [34] and supported by CSF biomarker data for tau, p-tau181, and Aβ42. This will include evaluation for other disorders which could significantly contribute to cognitive impairment, and will also include results from ancillary structural imaging, neuropsychological testing, and FDG-PET imaging (when available) [34].

In individuals who meet standard criteria for dementia due to AD, the CDR will be used to determine the severity of dementia. A CDR designation of 1, 2, and 3 denotes mild, moderate, and severe AD dementia, respectively [35].

Normal cognition will be defined as cognitive performance on detailed neuropsychological assessments that falls within 1 SD of age-, gender-, and education-matched norms in all cognitive domains, and no subjective report of cognitive decline from an individual’s baseline (ie, CDR 0). CSF biomarker data will be used to differentiate cognitively normal controls who have no biomarker evidence of AD pathology (ie, those with normal CSF tau, p-tau181 and Aβ42 levels: CSF tau<350 pg/ml, p-tau181<50 pg/ml, and Aβ42>500 pg/ml) from cognitively normal controls who have biomarker evidence of AD pathology (ie, those with the CSF biomarker phenotype of AD: CSF tau≥350 pg/ml, p-tau181≥50 pg/ml, and Aβ42≤500 pg/ml) at the time of data analysis. These cutoff values are based on the CSF biomarker levels that provided the highest diagnostic accuracy (ie, combination of sensitivity and specificity as measured by the area under the curve [AUC] for the receiver operating characteristic [ROC] curves) in differentiating individuals with a clinical diagnosis of AD from cognitively normal controls in previous longitudinal studies of healthy aging and dementia at the Washington University’s Knight Alzheimer’s Disease Research Center [36].

### Clinical Assessments

Clinical assessments will be performed by neurologists and nurse practitioners in the Cognitive Neurology clinic of the Ohio State University. Clinical assessments will include a detailed review of the history of present illness, past medical, surgical, social, and family history, medications, allergies, and a detailed physical and neurological exam.

### Neuropsychological Assessments

Neuropsychological assessments will be performed by experienced neuropsychometricians, and will include the following tests [37]: (1) Associate learning subtest of the Wechsler memory scale-IV [WMS-IV] [38]; (2) WMS-IV Logical Memory (I and II) [38]; (3) Hopkins Verbal Learning Test-Revised [39]; (4) Information subtest from the Wechsler adult intelligence scale-IV [WAIS-IV] [40]; (5) Boston naming test (short version) [41]; (6) animal fluency test [42]; (7) WMS-IV mental control (symbol span) [38]; (8) digit span forward and digit span backward (WAIS-IV) [40]; (9) letter fluency for F and S [43]; (10) block design (WAIS-IV) [40]; (11) digit symbol substitution tests [44]; (12) trail making tests A and B [45]; (13) the CDR [46]; (14) the Mini Mental Status Examination (MMSE) [47]; (15) the Self-Administered Gerocognitive Examination (SAGE) [48]; (16) the Geriatric Depression Scale (GDS) [49]; (17) the behavioral component of the Neuropsychiatric Inventory (NPI) [50]; and (18) the Functional Activity Questionnaire [51].

### Plasma Collection and Apolipoprotein E Genotyping

A total of 10 ml of blood will be obtained from each participant, collected in EDTA tubes, aliquoted, and frozen at –80°C. APOE genotyping will be performed using real-time PCR (polymerase chain reaction) on an Applied Biosystems 7900HT Real-Time PCR machine using the TaqMan SNP (single nucleotide polymorphism) Genotyping Assay (Applied Biosystems) for rs429358 and rs7412 as described [52].

### Lumbar Puncture

Each participant will undergo one LP within 4 months of the clinical and neuropsychological assessments. A total of 20-25 ml will be obtained from each participant in the lateral decubitus position under sterile conditions, collected in sterile polypropylene tubes, centrifuged, aliquoted and placed on dry ice. CSF aliquots will be stored at –80°C, then thawed and centrifuged prior to analysis. CSF analyses of tau, p-tau181, and Aβ42 levels will be performed using the Innotest enzyme-linked immunoassay (Fujirebio, formerly Innogenetics) as described [7]. CSF analyses for Ng and SNAP-25 levels will be performed using a single molecule counting chemiluminescence assay (Erenna, Singulex) as described [7,53].

### Magnetic Resonance Imaging

#### Structural Magnetic Resonance Imaging

Structural MRI data will be collected using a Siemens 3.0 Tesla Prisma scanner (Siemens, Erlangen, Germany). One to four T_1_-weighted sagittal magnetization-prepared rapid gradient-echo (MP-RAGE) scans will be acquired from each participant. Image processing will be performed as described [54,55]. High resolution, three-dimensional anatomic images will be acquired using the MP-RAGE sequence (TE [echo time]=2.45 milliseconds [ms]; TR [time to repetition]=2500 ms; inversion time=1060 ms; flip angle=8 degrees; slice thickness=1.0 mm; field of view [FOV]=256 mm; matrix size=256 × 256; and a resolution of 1 × 1 × 1 mm). Foam padding will be used to reduce head movement within the coil.

Whole brain volume will be obtained using freely available Freesurfer 5.0 software [56,57], with segmentation classifying each voxel of the MRI image as CSF, gray matter, or white matter. Normalized whole brain volumes (nWBVs) will be computed as the proportion of all voxels occupied by gray and white matter (equivalent to 100% minus the percentage of CSF) voxels, yielding a unit that represents the proportion of estimated total intracranial volume (ICV).

#### Functional Magnetic Resonance Imaging

Whole brain resting state and task activated fMRI will be conducted on a Siemens 3.0 Tesla Prisma scanner equipped with a 32-channel head array coil. Echo planar images will be collected using a pulse sequence (TE=28 ms; flip angle=60 degrees; FOV=240 mm; and matrix size=72 x 80). Forty-five contiguous axial 3-mm-thick slices will be selected to provide coverage of the entire brain (voxel size=3 × 3 × 3 mm). The TR will be 1 second.

Functional images will be preprocessed and registered using fMRI of the Brain Software Library techniques [58]. Data will be preprocessed according to a standard functional analysis pipeline employing motion correction, spatial smoothing using an *a priori* determined full width half maximum Gaussian smoothing kernel, high-pass temporal filtering at 0.01 hertz (Hz) to remove any low frequency noise from scanner drift or participant-related artifacts, brain extraction, and nonlinear spatial registration to optimize individual anatomical localization. FC within the DMN and semantic memory networks will be examined using a region of interest (ie, seed) model. Following the pipeline employed in previous studies [59-61], we will choose the left and right precuneus as seeds for the DMN and the semantic memory networks to create voxel-wise partial correlation maps, representing a correlation between the timeseries of the seed and that of every voxel in the brain. These individual level maps will then be forwarded separately to higher-level analyses, whereby intersubject variability will be treated as a random variable. These higher-level analyses will compare differences in FC of the DMN and the semantic memory networks between individuals with MCI or mild dementia due to AD (CDR 0.5-1) and cognitively normal controls (CDR 0). All maps will be thresholded at *z*=2.33 (*P*<.01) and a cluster threshold of *P*<.05 to correct for multiple comparisons. For each participant, the functional imaging data will first be registered to the participant’s high-resolution MP-RAGE, followed by registration to the Montreal Neurological Institute template [62]. Nonlinear transformations will be employed for all registrations to account for the significant heterogeneity in brain structure observed in clinical populations.

#### Semantic Memory Task

The task activated fMRI will be obtained during performance of the FNDT, a semantic memory task which consists of the presentation of 30 highly recognizable famous names and 30 unfamiliar names. Accuracy and reaction time will be recorded. The use of a semantic memory task offers several advantages over episodic memory tasks in MCI and mild AD dementia. In contrast to episodic memory tasks, which may be impaired with healthy aging, semantic memory tasks remain relatively intact in healthy elderly individuals but are impaired in the presence of AD pathology [32]. Furthermore, semantic memory tasks are easier and less frustrating for the elderly to perform, thereby allowing for more accuracy in interpreting test results by eliminating confounding effects of increased mental effort on fMRI signal. The FNDT has been successfully applied in previous fMRI studies of MCI and AD dementia [32].

### Statistical Analysis

Student’s *t* tests, chi-square (*X*^2^) analyses, and analysis of covariance (ANCOVA) will examine differences in demographic, clinical, neuropsychological, CSF biomarker, and FC measures between the study groups (SPSSv15, SPSS, IL). Partial correlation analyses and linear regression models will examine associations between CSF biomarker levels and FC measures, adjusting for age, gender, and the APOE4 genotype (SPSSv15, SPSS, IL). Bootstrap analyses will compare correlations between CSF biomarker measures (individually or as combinations of markers, using principal components analysis) and FC in the DMN and semantic memory networks in AD and controls (R Statistical Software).

### Outcome Measures

The main outcome measures of the study include CSF biomarker measurements (CSF Ng, SNAP-25, tau, p-tau181, and Aβ42 levels in pg/ml) and functional imaging measures including FC of the left and right precuneus seeds (ie, correlation between the timeseries of each of the left and right precuneus seeds and that of every voxel in the brain represented by voxel-wise partial correlation maps) during resting state and the performance of the semantic memory task (ie, FNDT). Analyses will be adjusted for covariates including age, gender, and the APOE4 genotype.

## Results

A total of 35 potential participants underwent initial screening for the study. Of those, 22 participants met the eligibility criteria and were subsequently enrolled in the study. Three participants were lost to follow-up during the study period. Therefore, a total of 19 participants (n=12 cognitively normal controls and n=7 participants with a clinical diagnosis of MCI/mild dementia due to AD) are currently enrolled in this study. Participant enrollment and study procedures are currently underway.

## Discussion

The main purpose of this study is to examine cross-sectional associations between CSF markers of synaptic injury (Ng and SNAP-25) and FC in the default mode and semantic memory networks using 3T-functional MRI in early symptomatic AD (MCI and mild dementia due to AD; CDR 0.5 and 1, respectively; n=20) and cognitively normal controls (CDR 0; n=20). To our knowledge, this is the first study to investigate associations between CSF markers of synaptic injury and FC in early symptomatic AD and healthy controls. We have previously demonstrated correlations of CSF Ng levels with whole brain and regional atrophy in AD [7], however, we are not aware of any studies which have investigated correlations between CSF Ng or SNAP-25 levels and functional imaging measures in AD or healthy aging. Furthermore, this will be the first study to examine associations between CSF biomarkers of AD pathology (including CSF markers of synaptic injury) and FC during the performance of a semantic memory task (ie, FNDT) which can be reliably performed by individuals with early symptomatic AD (CDR 0.5-1).

We hypothesize that higher CSF Ng and SNAP-25 levels (ie, reflective of more severe synaptic injury) will be associated with lower FC of the left and right precuneus seeds during resting state and the performance of the FNDT in individuals with MCI and mild AD dementia. Conversely, we hypothesize that no significant correlations between CSF Ng and SNAP-25 levels and FC of the left and right precuneus seeds will be observed during resting state or the performance of the FNDT in cognitively normal controls.

The identification of CSF biomarkers that reflect functional alterations in neural networks affected by early AD pathology (ie, default mode and semantic memory networks) will shed light on the potential utility of synaptic proteins as CSF surrogates of functional connectivity within neural networks and provide useful information regarding their value as potential outcome measures or stratification tools in clinical trials of AD therapeutics. CSF markers of synaptic injury may provide valuable tools to monitor disease progression, target engagement, and response to disease modifying therapies which target different pathological substrates of AD independently of changes to amyloid or tau pathology. Imaging methods that utilize amyloid binding ligands do not reliably reflect soluble Aβ species, which contribute significantly to synaptic damage and cognitive impairment in AD. Therefore, synaptic markers may offer useful measures for disease outcomes and therapeutic response at an earlier stage, and to a better degree, than CSF or imaging markers of amyloid or tau pathology. Importantly, this study will provide insight into the molecular mechanisms that underly the radiologic correlates of neural activity in different stages of disease and will improve our understanding of the dynamic interface between CSF and imaging surrogates of synaptic activity in the presence and absence of AD pathology.

## Appendix

Multimedia Appendix 1 Checklist for magnetic resonance imaging and lumbar puncture contraindications.

## Abbreviations

AD: Alzheimer disease
ANCOVA: analysis of covariance
APOE: apolipoprotein E
AUC: area under the curve
CBC: complete blood count
CDR: Clinical Dementia Rating
CHEI: cholinesterase-inhibitors
CSF: cerebrospinal fluid
CT: computed tomography
DMN: default mode network
FC: functional connectivity
FDG-PET: 18F-fludeoxy-glucose positron emission tomography
fMRI: functional magnetic resonance imaging
FNDT: Famous Name Discrimination Task
FOV: field of view
GDS: Geriatric Depression Scale
Hz: hertz
ICV: intracranial volume
INR: international normalized ratio
LP: lumbar puncture
MCI: mild cognitive impairment
MMSE: Mini Mental Status Examination
MP-RAGE: magnetization-prepared rapid gradient-echo
MRI: magnetic resonance imaging
Ng: neurogranin
NIA-AA: National Institute on Aging and Alzheimer’s Association
NPI: Neuropsychiatric Inventory
nWBV: normalized whole brain volume
PCR: polymerase chain reaction
PT: prothrombin time
p-tau181: tau phosphorylated at threonine 181
PTT: partial thromboplastin time
ROC: receiver operating characteristic
SAGE: Self-Administered Gerocognitive Examination
SNAP-25: synaptosome–associated protein-25
SNP: single nucleotide polymorphism
TE: echo time
TR: time to repetition
WAIS-IV: Wechsler adult intelligence scale-IV
WMS-IV: Wechsler memory scale-IV

## References

[1] Price JL, Morris JC (1999). Tangles and plaques in nondemented aging and "preclinical" Alzheimer's disease. Ann Neurol.

[2] Price JL, Ko AI, Wade MJ, Tsou SK, McKeel DW, Morris JC (2001). Neuron number in the entorhinal cortex and CA1 in preclinical Alzheimer disease. Arch Neurol.

[3] Jack CR, Knopman DS, Jagust WJ, Shaw LM, Aisen PS, Weiner MW, Petersen RC, Trojanowski JQ (2010). Hypothetical model of dynamic biomarkers of the Alzheimer's pathological cascade. Lancet Neurol.

[4] DeKosky ST, Scheff SW (1990). Synapse loss in frontal cortex biopsies in Alzheimer's disease: correlation with cognitive severity. Ann Neurol.

[5] Terry RD, Masliah E, Salmon DP, Butters N, DeTeresa R, Hill R, Hansen LA, Katzman R (1991). Physical basis of cognitive alterations in Alzheimer's disease: synapse loss is the major correlate of cognitive impairment. Ann Neurol.

[6] Laterza OF, Modur VR, Crimmins DL, Olander JV, Landt Y, Lee J, Ladenson JH (2006). Identification of novel brain biomarkers. Clin Chem.

[7] Tarawneh R, D'Angelo G, Crimmins D, Herries E, Griest T, Fagan AM, Zipfel GJ, Ladenson JH, Morris JC, Holtzman DM (2016). Diagnostic and Prognostic Utility of the Synaptic Marker Neurogranin in Alzheimer Disease. JAMA Neurol.

[8] Watson JB, Szijan I, Coulter PM (1994). Localization of RC3 (neurogranin) in rat brain subcellular fractions. Brain Res Mol Brain Res.

[9] Brinkmalm A, Brinkmalm G, Honer WG, Frölich L, Hausner L, Minthon L, Hansson O, Wallin A, Zetterberg H, Blennow K, Öhrfelt A (2014). SNAP-25 is a promising novel cerebrospinal fluid biomarker for synapse degeneration in Alzheimer's disease. Mol Neurodegener.

[10] Díez-Guerra FJ (2010). Neurogranin, a link between calcium/calmodulin and protein kinase C signaling in synaptic plasticity. IUBMB Life.

[11] Zhong L, Gerges NZ (2012). Neurogranin targets calmodulin and lowers the threshold for the induction of long-term potentiation. PLoS One.

[12] Wang JH, Kelly PT (1995). Postsynaptic injection of CA2+/CaM induces synaptic potentiation requiring CaMKII and PKC activity. Neuron.

[13] Miyakawa T, Yared E, Pak JH, Huang FL, Huang KP, Crawley JN (2001). Neurogranin null mutant mice display performance deficits on spatial learning tasks with anxiety related components. Hippocampus.

[14] Pak J, Huang F L, Li J, Balschun D, Reymann K G, Chiang C, Westphal H, Huang K P (2000). Involvement of neurogranin in the modulation of calcium/calmodulin-dependent protein kinase II, synaptic plasticity, and spatial learning: a study with knockout mice. Proc Natl Acad Sci U S A.

[15] Huang K, Huang FL, Jäger T, Li J, Reymann KG, Balschun D (2004). Neurogranin/RC3 enhances long-term potentiation and learning by promoting calcium-mediated signaling. J Neurosci.

[16] Gerendasy D (1999). Homeostatic tuning of Ca2+ signal transduction by members of the calpacitin protein family. J Neurosci Res.

[17] Gerendasy DD, Sutcliffe JG (1997). RC3/neurogranin, a postsynaptic calpacitin for setting the response threshold to calcium influxes. Mol Neurobiol.

[18] Jahn R, Südhof TC (1999). Membrane fusion and exocytosis. Annu Rev Biochem.

[19] Hess DT, Slater TM, Wilson MC, Skene JH (1992). The 25 kDa synaptosomal-associated protein SNAP-25 is the major methionine-rich polypeptide in rapid axonal transport and a major substrate for palmitoylation in adult CNS. J Neurosci.

[20] Portelius E, Zetterberg Henrik, Skillbäck Tobias, Törnqvist Ulrika, Andreasson Ulf, Trojanowski John Q, Weiner Michael W, Shaw Leslie M, Mattsson Niklas, Blennow Kaj, Alzheimer’s Disease Neuroimaging Initiative (2015). Cerebrospinal fluid neurogranin: relation to cognition and neurodegeneration in Alzheimer's disease. Brain.

[21] Zhang H, Therriault J, Kang MS, Ng KP, Pascoal TA, Rosa-Neto P, Gauthier S, Alzheimer’s Disease Neuroimaging Initiative (2018). Cerebrospinal fluid synaptosomal-associated protein 25 is a key player in synaptic degeneration in mild cognitive impairment and Alzheimer's disease. Alzheimers Res Ther.

[22] Sutphen CL, McCue L, Herries EM, Xiong C, Ladenson JH, Holtzman DM, Fagan AM, ADNI (2018). Longitudinal decreases in multiple cerebrospinal fluid biomarkers of neuronal injury in symptomatic late onset Alzheimer's disease. Alzheimers Dement.

[23] Mevel K, Chételat G, Eustache F, Desgranges B (2011). The default mode network in healthy aging and Alzheimer's disease. Int J Alzheimers Dis.

[24] Greicius M, Srivastava Gaurav, Reiss Allan L, Menon Vinod (2004). Default-mode network activity distinguishes Alzheimer's disease from healthy aging: evidence from functional MRI. Proc Natl Acad Sci U S A.

[25] Greicius MD, Menon V (2004). Default-mode activity during a passive sensory task: uncoupled from deactivation but impacting activation. J Cogn Neurosci.

[26] Greicius MD, Supekar K, Menon V, Dougherty RF (2009). Resting-state functional connectivity reflects structural connectivity in the default mode network. Cereb Cortex.

[27] Hafkemeijer A, van der Grond J, Rombouts SARB (2012). Imaging the default mode network in aging and dementia. Biochim Biophys Acta.

[28] van den Heuvel Martijn P, Hulshoff Pol Hilleke E (2010). Exploring the brain network: a review on resting-state fMRI functional connectivity. Eur Neuropsychopharmacol.

[29] Rogers SL, Friedman RB (2008). The underlying mechanisms of semantic memory loss in Alzheimer's disease and semantic dementia. Neuropsychologia.

[30] Binder JR, Desai RH, Graves WW, Conant LL (2009). Where is the semantic system? A critical review and meta-analysis of 120 functional neuroimaging studies. Cereb Cortex.

[31] Binder JR, Desai RH (2011). The neurobiology of semantic memory. Trends Cogn Sci.

[32] Sugarman MA, Woodard JL, Nielson KA, Seidenberg M, Smith JC, Durgerian S, Rao SM (2012). Functional magnetic resonance imaging of semantic memory as a presymptomatic biomarker of Alzheimer's disease risk. Biochim Biophys Acta.

[33] Albert MS, DeKosky ST, Dickson D, Dubois B, Feldman HH, Fox NC, Gamst A, Holtzman DM, Jagust WJ, Petersen RC, Snyder PJ, Carrillo MC, Thies B, Phelps CH (2011). The diagnosis of mild cognitive impairment due to Alzheimer's disease: recommendations from the National Institute on Aging-Alzheimer's Association workgroups on diagnostic guidelines for Alzheimer's disease. Alzheimers Dement.

[34] McKhann GM, Knopman DS, Chertkow H, Hyman BT, Jack CR, Kawas CH, Klunk WE, Koroshetz WJ, Manly JJ, Mayeux R, Mohs RC, Morris JC, Rossor MN, Scheltens P, Carrillo MC, Thies B, Weintraub S, Phelps CH (2011). The diagnosis of dementia due to Alzheimer's disease: recommendations from the National Institute on Aging-Alzheimer's Association workgroups on diagnostic guidelines for Alzheimer's disease. Alzheimers Dement.

[35] Morris JC, Blennow K, Froelich L, Nordberg A, Soininen H, Waldemar G, Wahlund L, Dubois B (2014). Harmonized diagnostic criteria for Alzheimer's disease: recommendations. J Intern Med.

[36] Tarawneh R, D'Angelo G, Macy E, Xiong C, Carter D, Cairns NJ, Fagan AM, Head D, Mintun MA, Ladenson JH, Lee J, Morris JC, Holtzman DM (2011). Visinin-like protein-1: diagnostic and prognostic biomarker in Alzheimer disease. Ann Neurol.

[37] Tarawneh R, Lee J, Ladenson JH, Morris JC, Holtzman DM (2012). CSF VILIP-1 predicts rates of cognitive decline in early Alzheimer disease. Neurology.

[38] Wechsler D (1945). A Standardized Memory Scale for Clinical Use. The Journal of Psychology.

[39] Benedict R, Schretlen D, Groninger L, Brandt J (2010). Hopkins Verbal Learning Test – Revised: Normative Data and Analysis of Inter-Form and Test-Retest Reliability. The Clinical Neuropsychologist.

[40] Patterson J, Kreutzer J.S., DeLuca J, Caplan B (2011). Verbal Fluency in Wechsler Adult Intelligence Scale. Encyclopedia Of Clinical Neuropsychology.

[41] Mack W, Freed D M, Williams B W, Henderson V W (1992). Boston Naming Test: shortened versions for use in Alzheimer's disease. J Gerontol.

[42] Sager MH, Hermann Bruce P, La Rue Asenath, Woodard John L (2006). Screening for dementia in community-based memory clinics. WMJ.

[43] Patterson J, Kreutzer JS, DeLuca J, Caplan B (2011). Verbal Fluency. Encyclopedia Of Clinical Neuropsychology.

[44] Rosano C, Perera S, Inzitari M, Newman AB, Longstreth WT, Studenski S (2016). Digit Symbol Substitution test and future clinical and subclinical disorders of cognition, mobility and mood in older adults. Age Ageing.

[45] Tombaugh TN (2004). Trail Making Test A and B: normative data stratified by age and education. Arch Clin Neuropsychol.

[46] Morris JC (1993). The Clinical Dementia Rating (CDR): current version and scoring rules. Neurology.

[47] Folstein MF, Folstein SE, McHugh PR (1975). "Mini-mental state". A practical method for grading the cognitive state of patients for the clinician. J Psychiatr Res.

[48] Scharre D, Chang Shu-Ing, Murden Robert A, Lamb James, Beversdorf David Q, Kataki Maria, Nagaraja Haikady N, Bornstein Robert A (2010). Self-administered Gerocognitive Examination (SAGE): a brief cognitive assessment Instrument for mild cognitive impairment (MCI) and early dementia. Alzheimer Dis Assoc Disord.

[49] Yesavage J, Sheikh Ji (2008). 9/Geriatric Depression Scale (GDS). Clinical Gerontologist.

[50] Cummings J (1997). The Neuropsychiatric Inventory: assessing psychopathology in dementia patients. Neurology.

[51] Pfeffer RI, Kurosaki TT, Harrah CH, Chance JM, Filos S (1982). Measurement of functional activities in older adults in the community. J Gerontol.

[52] Damoiseaux JS, Seeley WW, Zhou J, Shirer WR, Coppola G, Karydas A, Rosen HJ, Miller BL, Kramer JH, Greicius MD, Alzheimer's Disease Neuroimaging Initiative (2012). Gender modulates the APOE ε4 effect in healthy older adults: convergent evidence from functional brain connectivity and spinal fluid tau levels. J Neurosci.

[53] Sutphen C, Herries E, Crimmins D, Schindler Se, Ladenson Jh, Morris Jc, Holtzman Dm, Fagan Am (2016). Emerging CSF biomarkers of neuroinflammation, neuronal injury and synaptic integrity in the ADNI cohort. Alzheimer's & Dementia.

[54] Buckner RL, Head D, Parker J, Fotenos AF, Marcus D, Morris JC, Snyder AZ (2004). A unified approach for morphometric and functional data analysis in young, old, and demented adults using automated atlas-based head size normalization: reliability and validation against manual measurement of total intracranial volume. Neuroimage.

[55] Head D, Snyder AZ, Girton LE, Morris JC, Buckner RL (2005). Frontal-hippocampal double dissociation between normal aging and Alzheimer's disease. Cereb Cortex.

[56] Zhang Y, Brady M, Smith S (2001). Segmentation of brain MR images through a hidden Markov random field model and the expectation-maximization algorithm. IEEE Trans Med Imaging.

[57] Smith SM (2002). Fast robust automated brain extraction. Hum Brain Mapp.

[58] Smith SM, Zhang Y, Jenkinson M, Chen J, Matthews PM, Federico A, De Stefano N (2002). Accurate, robust, and automated longitudinal and cross-sectional brain change analysis. Neuroimage.

[59] Prakash RS, Patterson B, Janssen A, Abduljalil A, Boster A (2011). Physical activity associated with increased resting-state functional connectivity in multiple sclerosis. J Int Neuropsychol Soc.

[60] Shaurya Prakash R, De Leon AA, Klatt M, Malarkey W, Patterson B (2013). Mindfulness disposition and default-mode network connectivity in older adults. Soc Cogn Affect Neurosci.

[61] Fountain-Zaragoza S, Samimy S, Rosenberg MD, Prakash RS (2019). Connectome-based models predict attentional control in aging adults. Neuroimage.

[62] Lancaster JL, Tordesillas-Gutiérrez D, Martinez M, Salinas F, Evans A, Zilles K, Mazziotta JC, Fox PT (2007). Bias between MNI and Talairach coordinates analyzed using the ICBM-152 brain template. Hum Brain Mapp.

