# Supplementary material for: Cerebrospinal Fluid Markers of Synaptic Injury and Functional Connectivity in Alzheimer Disease: Protocol for a Cross-Sectional Study
Source: JMIR Res Protoc. 2019 Jul 17;8(7):e14302. doi: 10.2196/14302 (PMC6668296; doi:10.2196/14302)
Supplement: Multimedia Appendix 1 [file resprot_v8i7e14302_app1.docx]

SUPPLEMENT

Contraindications to Magnetic Resonance Imaging:

*Absolute contraindications*:

- Permanent pacemakers and Implantable Cardiac Defibrillators (ICD)
- Pulmonary artery monitoring and thermodilution devices (i.e. Swan-Ganz catheters)
- Temporary cardiac pacemaker (i.e. external pulse generator or temporary pacing leads)
- Hemodynamic monitoring and support devices (i.e. intra-aortic balloon pump and ventricular assist devices)
- Retained transvenous pacemaker and defibrillator leads
- Neural stimulator systems
- Cochlear implants
- Ferromagnetic aneurysm clips
- Transdermal patches that contain aluminum or other metals
- Metallic bodies in the eyes

*Relative contraindications and precautions:*

Safety of MRI in these cases will be determined by the supervising radiologist according to the type of device and individual participant factors (in accordance with clinical practice guidelines followed by Radiology in these situations). In these cases, structural and functional MRIs will only be performed if the radiologist and study investigators determine that these devices are MRI-compatible, and that performance of the MRI poses minimal to no risk to the participant.

- Metal hip replacements, sutures or foreign bodies in other sites
- Cardiac closure and occluding devices
- Coronary artery stents
- Loop recorders (event monitor)
- Cardiac embolization coils
- Inferior Vena Cava (IVC) filters
- Peripheral vascular stents

Contraindications to Lumbar puncture:

- Allergy to lidocaine or other local anesthetics
- Possible raised intracranial pressure
- Thrombocytopenia (< 100,000/µL), prothrombin time (PT) ≥ 15 seconds, international normalized ratio (INR) ≥ 1.4, or partial thromboplastin time (PTT) ≥ 40
- Treatment with oral or intravenous anticoagulants (e.g. heparin, low-molecular weight heparins, warfarin, dabigatran, rivaroxaban, apixaban)
- Suspected spinal epidural abscess
- Active skin or subcutaneous infection at the site of the procedure
